# Enhancing Therapeutic Efficacy of Oncolytic Herpes Simplex Virus with MEK Inhibitor Trametinib in Some BRAF or KRAS-Mutated Colorectal or Lung Carcinoma Models

**DOI:** 10.3390/v13091758

**Published:** 2021-09-03

**Authors:** XuSha Zhou, Jing Zhao, Jian V. Zhang, Yinglin Wu, Lei Wang, Xiaoqing Chen, Dongmei Ji, Grace Guoying Zhou

**Affiliations:** 1 Shenzhen International Institute for Biomedical Research, Shenzhen 518110, China; zhouxusha1900@163.com (X.Z.); zhaojingnh2005@163.com (J.Z.); chennancy@siitm.org.cn (X.C.); 2 Center for Energy Metabolism and Reproduction, Institute of Biomedicine and Biotechnology, Shenzhen Institutes of Advanced Technology, Chinese Academy of Sciences, Shenzhen 518055, China; 3 Department of Immunology, School of Basic Medical Sciences, Guangzhou Medical University, Guangzhou 511436, China; wu_ylin@163.com (Y.W.); kathrynchouchou@163.com (L.W.); 4 Department of Medical Oncology, Shanghai Cancer Center and Shanghai Medical College, Fudan University, Shanghai 200032, China; jidongmei2000@hotmail.com

**Keywords:** oHSV, MEKi trametinib, BRAF, KRAS, antitumor

## Abstract

Oncolytic virus (OV) as a promising therapeutic agent can selectively infect and kill tumor cells with naturally inherited or engineered properties. Considering the limitations of OVs monotherapy, combination therapy has been widely explored. MEK inhibitor (MEKi) Trametinib is an FDA-approved kinase inhibitor indicated for the treatment of tumors with BRAF V600E or V600K mutations. In this study, the oncolytic activity in vitro and anti-tumor therapeutic efficacy in vivo when combined with oHSV and MEKi Trametinib were investigated. We found: (1) Treatment with MEKi Trametinib augmented oHSV oncolytic activity in BRAF V600E-mutated tumor cells. (2) Combination treatment with oHSV and MEKi Trametinib enhanced virus replication mediated by down-regulation of STAT1 and PKR expression or phosphorylation in BRAF V600E-mutated tumor cells as well as BRAF wt/KRAS-mutated tumor cells. (3) A remarkably synergistic therapeutic efficacy was shown in vivo for BRAF wt/KRAS-mutated tumor models, when a combination of oHSV including PD-1 blockade and MEK inhibition. Collectively, these data provide some new insights for clinical development of combination therapy with oncolytic virus, MEK inhibition, and checkpoint blockade for BRAF or KRAS-mutated tumors.

## 1. Introduction

Oncolytic virus (OV) as a promising therapeutic agent can selectively infect and destroy tumor cells with naturally inherited or engineered properties [1]. However, therapeutic responses with OVs monotherapy remains limited to a few patients, and the limitations include physical and immunological barriers that impede OV delivery, replication, and spread within tumor tissues [2]. Thus, development of combination therapy with therapeutic modalities has been widely explored in antitumor research and clinical trials. For example, the combination of OVs with other immunotherapies, especially immune checkpoint blockade (ICB) can exert synergistic effect to increase therapeutic effects against tumors [1,3]. The pharmacoviral approaches combining OVs with molecular therapeutics targeting kinases commonly linked to cancer development also showed remarkably synergistic responses during tumor treatment [4].

The mitogen-activated protein kinase (MAPK) cascade (RAS/RAF/MEK/ERK), which is one of the most dysregulated pathways in human cancers, regulates multiple critical cellular functions including proliferation, growth, survival and motility [5,6]. In the RAS/RAF/MEK/ERK signaling cascade, MEK is activated through phosphorylation by its upstream MAPK kinase kinase (known as RAF) and RAF is activated when the small GTP-binding protein, RAS, is in its GTP-bound form [7]. The mutations involving the GTP-ase RAS protein family and its downstream BRAF lead to loss of cell cycle regulation at key checkpoints, which are the main driver mutations for several tumor types, e.g., colorectal carcinogenesis [8].

Pre-clinical studies have shown that MAPK pathway inhibition synergizes with oHSV infection in melanoma cells. Bommareddy et al. reported that the T-VEC/ MEK inhibitor (MEKi) regime increased viral replication and apoptosis within melanoma cells in vitro, furthermore, reduced tumor growth in vivo [9]. Yoo et al. demonstrated that pre-treatment with the MEKi can enhance oHSV replication within glioma cells by suppressing macrophage-mediated apoptosis of tumor cells [10]. The MEKi Trametinib used in these studies, is an FDA-approved kinase inhibitor indicated for the treatment of melanoma, non-small cell lung cancer and thyroid cancer with BRAF V600E or V600K mutations [11].

Inhibition of MEK was presumed to be particularly efficacious in cancers harboring a RAS or RAF mutation, leading to overactivation of the MAPK pathway [12]. In BRAF V600E–mutant melanoma, MEK inhibition was shown to activate caspase-3 through BIM-EL and BMF-mediated mitochondrial depolarization, which leads to apoptosis in the tumor cells [13]. In addition, MEK inhibition can sensitize unresponsive KRAS-mutated tumors to immunotherapy [14]. Here, studies in this manuscript tested whether the combination with oHSV and MEKi Trametinib can enhance oncolytic virus replication and oncolytic activity in BRAF-mutated or RAS-mutated tumor in vitro as well as the antitumor efficacy in vivo.

## 2. Materials and Methods

### 2.1. Cell Lines and Virus

Vero cells and Widr cells were obtained from the American Type Culture Collection and were routinely cultured in DMEM (Life Technologies) supplemented with 5% (vol/vol) newborn bovine serum (NBCS) or 10% (vol/vol) fetal bovine serum (FBS, Gibco), respectively. Caco-2 cells were purchased from ATCC and maintained in MEM-EBSS (HYCLONE) supplemented with 20% (vol/vol) FBS. HT29 cells were obtained from the Cell Bank of Sun Yat-sen University Experimental Animal Center and were cultured in McCoy’s 5A Medium Modified (ATCC) supplemented with 10% (vol/vol) FBS. 4T1(Mouse breast cancer cell), CT26 (Mouse colorectal carcinoma cell) and LLC (Mouse lung adenocarcinoma cell) were kindly provided by Stem Cell Bank, Chinese Academy of Sciences. 4T1 and CT26 were maintained in RPMI-1640 (Life Technologies), containing 10% (vol/vol) FBS and LLC was routinely cultured in DMEM (Life Technologies) supplemented with 10% (vol/vol) FBS (Gibco). All culture media were supplemented with 100 U/mL penicillin and 100 μg/mL streptomycin.

Oncolytic herpes virus (oHSV) T1012G and T3855 encoding murine IL-12 and PD-1 antibody was reported elsewhere [15].

### 2.2. Antibodies

Antibodies used in this study included p-ERK (Cat. #4370T, Cell Signaling Technology), ERK (Cat. #4695T, Cell Signaling Technology), p-STAT1 (Cat. #7649S, Cell Signaling Technology), STAT1 (Cat. #10144-2-AP, Proteintech), p-PKR (Cat. #ab32036, Abcam), Anti-phospho-PKR (Thr451) (07-886, Merck), PKR (Cat. #ab32506, Abcam), and anti-GAPDH (Cat. #2118S, Cell Signaling Technology).

### 2.3. Cell Counting Kit 8 Assay

The tumor cell killing activity was assessed using a CCK8 assay. Briefly, Widr, HT29 or Caco-2 cells were plated in 96-well plates at 5–8 × 10^3^ cells/well. MEK inhibitor (MEKi) Trametinib (MCE) was added after the cells had adhered overnight at a final concentration of 15 nM for 2 h. Then, cells were infected with T1012G for 48 h after pretreatment with MEKi. Cell viability was evaluated using Cell Counting Kit-8 (MCE) according to manufacturer’s instructions. The cell viability rate was calculated according to the following formula: Cell viability (100%) = ((A value of experimental wells)-(A value of the blank wells)/(A value of target cell wells)-(A value of the blank wells)) × 100%. The assay was performed in triplicate.

### 2.4. RNA Interference

The knockdown of STAT1 was achieved using siRNAs (GenePharma). The sequences of the siRNA were as follows:

si-STAT1: 5′-GAACCUGACUUCCAUGCGGTT-3′;

The None Target (NT) siRNA: 5′-UUCUCCGAACGUGUCACGUTT-3′.

The siRNA transfections were carried out using Lipofectamine 2000 (Invitrogen) according to manufacturer’s instructions.

### 2.5. RNA Extraction and Real-Time PCR

CT26 and 4T1 cell pellets after treatment were collected and total RNAs from cells were isolated using TRIzol reagent (Invitrogen). A total amount of 0.5 µg of RNA was reverse transcribed to cDNA using the PCR RT kit (TOYOBO) according to manufacturer’s instructions. The real-time PCR were performed using the StepOnePlus ™ Real-Time PCR System (Applied Biosystems) and the amplifications were performed using the SYBR Premix (TaKaRa). The mRNA expression levels were normalized by18S rRNA and analyzed using the 2 ^−ΔΔCT^ method.

Primers used for amplifications were as follows:

Mouse PKR-F 5′-CCTGAGCACAGCATGAGTGA-3′

Mouse PKR-R 5′-TACTCCACTCCGGTCACGAT-3′

Mouse STAT1-F 5′-GGAAGGGGCCATCACATTCA-3′

Mouse STAT1-R 5′-TACTTCCCAAAGGCGTGGTC-3′

18S-F 5′- CTCAACACGGGAAACCTCAC -3′

18S-R 5′- CGCTCCACCAACTAAGAACG -3′

### 2.6. Immunoblotting Assays

Caco-2, HT29, and Widr cell pellets were collected at the indicated time points post treatment. Total protein was extracted by RIPA Lysis Buffer (Beyotime Biotechnology) supplemented with 1 mM phenylmethylsulfonyl fluoride (PMSF). The isolated protein concentration was determined using BCA Protein Assay Kit (Beyotime Biotechnology). Twenty micrograms of the proteins were separated on a 10% SDS-polyacrylamide gel electrophoresis (PAGE) and transferred to polyvinylidene difluoride membranes (Millipore). The proteins were detected by incubation with indicated primary antibody, followed by horseradish peroxidase (HRP)-conjugated secondary antibody (Invitrogen) and the ECL reagent (Merck Millipore). Images were captured using a ChemiDoc Touch Imaging System (Bio-Rad) and processed using ImageLab software. The densities of corresponding bands were quantified using the ImageJ software.

### 2.7. Virus Infection and Titration

A total of 1 × 10^6^ cells were seeded onto six-well plates. After 24 h, the adherent cells were pretreated with DMSO or MEKi for 2 h and then exposed to 0.1 PFU T1012G or T3855 per cell. The cells were harvested at 48 h or 72 h after infection. Viral progeny was titrated on Vero cells after three freeze–thaw cycles.

### 2.8. Syngeneic Mouse Model and Tumor Treatment

Five-week-old female Balb/c mice and C57BL/6 mice were purchased from Charles River Laboratories (Beijing, China) and housed under specific pathogen-free (SPF) conditions. The syngeneic mice were Balb/c for 4T1 and CT26, C57BL/6 for LLC. The 4T1 and CT26 tumor models were generated by implantation of 1 × 10^6^ cells and 5 × 10^5^ cells subcutaneously into Balb/c mouse flanks, respectively. The LLC tumor model was generated by implantation of 1 × 10^6^ cells subcutaneously into C57BL/6 mouse flanks. In tumor treatment studies, BALB/c or C57BL/6 mice (n = 8 per group) were treated with oHSV T3855 (1 × 10^7^ PFU/mouse for 4T1 or LLC; 5 × 10^6^ PFU/mouse for CT26) or vehicle control via intratumoral injection on days 1, 8, 15, 22; MEKi (1.0 mg/kg) or vehicle control via oral gavage from days 1 to day 14. In addition, the combined group received equivalent doses of combination treatment with oHSV and MEKi. Tumor growth was measured in two dimensions, recording the greatest length and width using digital calipers. Tumor sizes were plotted as average size for each group. All institutional and national guidelines for the care and use of laboratory animals were followed.

### 2.9. Statistical Analysis

Data were expressed as mean ± standard deviation. Differences between datasets were assessed by student’s t-test (two-tailed) using the GraphPad Prism software. ^N.S^ *p* > 0.05, * *p* < 0.05, ** *p* < 0.01, *** *p* < 0.001.

## 3. Result

### 3.1. Treatment with MEK Inhibitor (MEKi) Trametinib Enhanced oHSV Replication and Tumor Cell Killing in BRAF V600E-Mutated Tumor Cells

**Figure 1:**
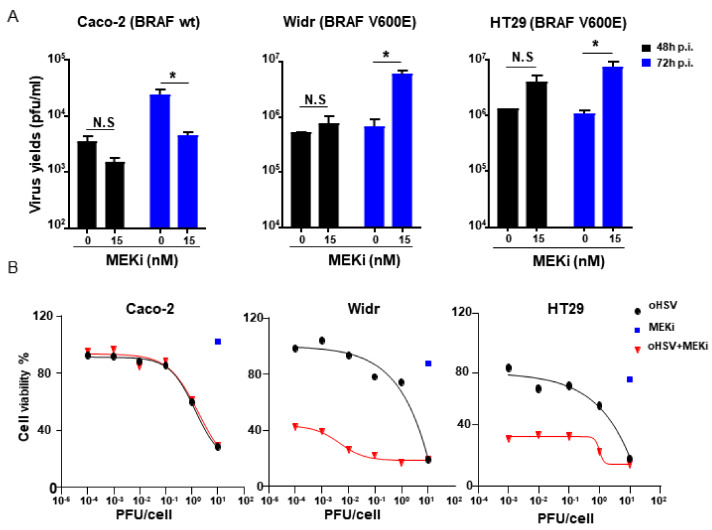
MEK inhibitor (MEKi) Trametinib treatment promoted oHSV replication and tumor cell killing in BRAF V600E-mutated tumor cells. (**A**) Caco-2, Widr, and HT29 cells were pretreated with 15 nM of Trametinib (MEKi) for 2 h and then infected with T1012G at 0.01 PFU/cell. The infected cell pellets were harvested at 48 and 72 h post-infection, respectively. The titration was measured by conventional plaque assay on Vero cells after three freeze–thaw cycles. (**B**) The tumor cell killing activity was assessed using a CCK8 assay. Caco-2, Widr, and HT29 cells were mock treated or pretreated with 15 nM of Trametinib (MEKi) for 2 h and then infected with T1012G at an indicated MOI or MEKi only treated. Cell viability of infected cells was measured with a CCK8 assay (mean ± SD) 48 h after infection. The assay was performed in triplicate. N.S. (not significant); *p* > 0.05, * *p* < 0.05.

As resistance to oHSV therapy occurred in many tumors, combination with other antitumor agents or therapies becomes a good strategy for augment of the oHSV antitumor therapeutic efficacy. In this study, MEKi Trametinib, an FDA-approved kinase inhibitor indicated for tumor treatment with BRAF V600E or V600K mutations was used as an antitumor agent to combine with oHSV T1012G. Then, the oHSV replication and tumor cell killing were investigated in both BRAF wild type (wt) (Caco-2 cell) and V600E-mutated tumor cells (Widr and HT29 cells) (Table 1). It is noted that the viral yields increased significantly in BRAF V600E-mutated tumor cells, about 8.6-fold for Widr cell and 6.7-fold for HT29 cell, respectively, when combination of oHSV and Trametinib at the concentration of 15 nM compared to oHSV alone at 72 hpi. In contrast, for BRAF wt Caco-2 cell, there is no increase but an around 5-fold decrease of the oHSV yields when combined treatment with Trametinib at 72 hpi (Figure 1A). At 48hpi, the oHSV replication displayed the similar pattern as 72 hpi, though without significant difference (Figure 1A). Compared with BRAF wt tumor cells, the oHSV infected tumor cells harboring BRAF V600E mutations exhibited increased cytotoxicity when pretreated with Trametinib (Figure 1B). Collectively, it suggested that pretreatment with MEKi Trametinib in BRAF V600E-mutated tumor cells facilitated oHSV replication and augmented virus oncolytic activity.

### 3.2. Treatment with Trametinib Induced Decreased Phosphorylation of STAT1 and PKR in BRAF V600E-Mutated Tumor Cells

**Figure 2:**
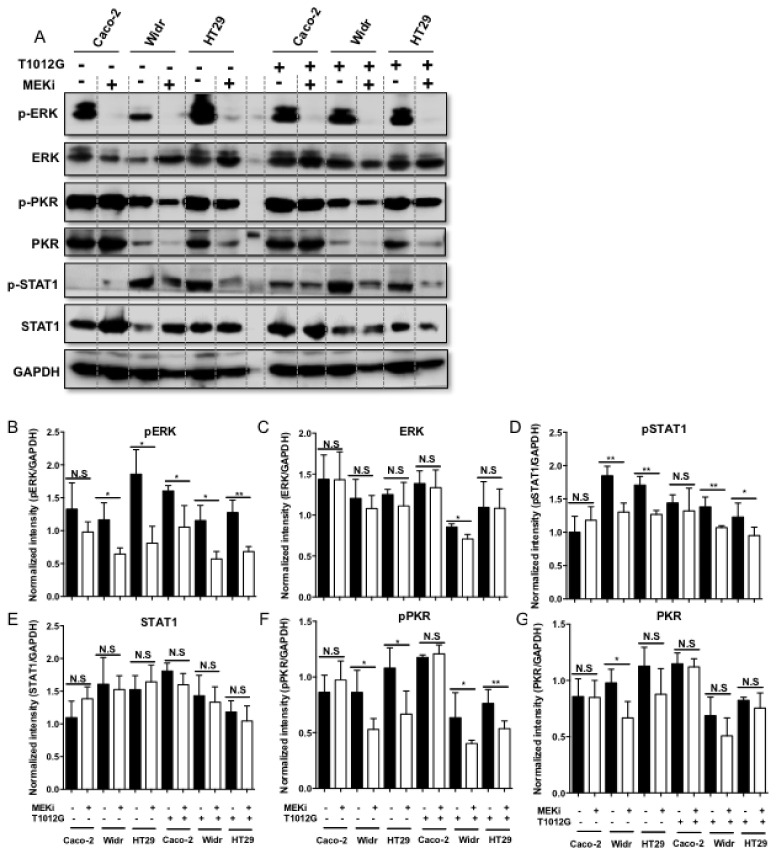
Down-regulation of STAT1 and PKR phosphorylation when combination of oHSV and MEKi in BRAF mutant cancer cells. (**A**) Caco-2, Widr, and HT29 cells were pretreated with 0.25 μM of Trametinib (MEKi) for 2 h and then mock infected or infected with 0.1 PFU/cell T1012G. The cell pellets were harvested at 24 h post-infection, respectively, and lysates were analyzed by Western blotting for the indicated proteins. (**B**–**G**). Quantification of the protein level of pERK, ERK, pSTAT1, STAT1, pPKR, and PKR. N.S. (not significant); *p* > 0.05, * *p* < 0.05, ** *p* < 0.01.

Next, investigation into the molecular basis behind how combination of MEKi and oHSV to enhance virus replication was performed. It has been reported that PKR as a host antiviral kinase can limit γ34.5 deleted oHSV late-gene expression and replication [16]. In addition to PKR, cells also express a diverse set of pattern recognition receptors (PRR) that detect pathogen-associated molecular patterns such as viral nucleic acids. Among them, IFN/STAT1 signaling modulators contributes to promote amplification of the antiviral response [17]. Thus, we sought to determine whether the expression and phosphorylation of PKR and STAT1 were down-regulated when treated with Trametinib alone or combined with oHSV. Firstly, we confirmed that MEKi alone induced significantly decreased levels of phosphorylated ERK (p-ERK) as well as ERK in both BRAF wt Caco-2 cell and V600E-mutated Widr and HT29 cells (Figure 2A–C). Similar decreased expression pattern of p-ERK and ERK was also observed in combination treatment with oHSV (Figure 2A–C). Then, we observed that the phosphorylation of PKR and STAT1 was significantly down-regulated with both MEKi alone and combination treatment in Widr and HT29 cells compared to Caco-2 cell (Figure 2D,F). For the total PKR and STAT1 expression, there was no significant difference either with MEKi alone or combination treatment in BRAF V600E-mutated tumor cells, with an exception that there is a remarkable decrease of the total PKR expression in Widr cells (Figure 2E,G). Taken together, it suggested that down-regulation of p-PKR and p-STAT1 induced by MEKi Trametinib may contribute to enhance oHSV replication in BRAF V600E-mutated cells.

### 3.3. Down-Regulation of STAT1 Supressed the Expression of PKR in BRAF V600E-Mutated Tumor Cells

**Figure 3:**
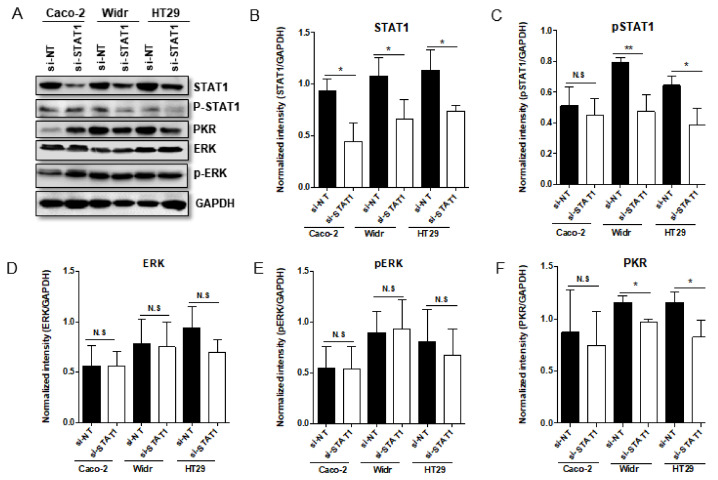
Knockdown of STAT1 in Caco-2, Widr and HT29 cells by siRNA. Panel (**A**) Caco-2, Widr, and HT29 cells were either transfected with 100 nM STAT1 siRNA or nontarget siRNA (siNT). The cells were harvested at 72 h after transfection. The proteins were electrophoretically separated in a 10% denaturing gel and reacted with indicated antibodies. Panel (**B**–**F**) Quantification of the protein level of STAT1, pSTAT1, ERK, pERK, and PKR in Caco-2, Widr, and HT29 cells when Knockdown of STAT1 by siRNA. N.S. (not significant); *p* > 0.05, * *p* < 0.05, ** *p* < 0.01.

To further confirm the possible relevance between STAT1 and PKR, knockdown of STAT1 by siRNA was performed in Caco-2, Widr, and HT29 cell. The results indicated that siRNA targeting STAT1 (si-STAT1) can efficiently down-regulate STAT1 protein expression compared to non-targeting siRNA (si-NT) transfection in all the cells tested (Figure 3A,B), whereas the efficient down-regulation of p-STAT1 was only shown in BRAF V600E-mutated Widr and HT29 cells (Figure 3A,C). Strikingly, the PKR levels were decreased under the condition with lower abundance of STAT1 in BRAF V600E-mutated Widr and HT29 cells while in BRAF wt Caco-2 cell, there is no significant decrease for PKR expression (Figure 3A,F). The difference of PKR expression induced by down-regulation of STAT1 between BRAF wt and V600E-mutated cells may explain the diversity of oHSV replication on these cells. Additional studies showed that down-regulation of STAT1 had no impact on ERK expression and ERK phosphorylation (Figure 3A,D,E).

### 3.4. MEKi Trametinib Treatment Promoted oHSV Replication by Down-Regulation of PKR and STAT1 mRNA Expression in KRAS Mutant Cancer Cells

**Figure 4:**
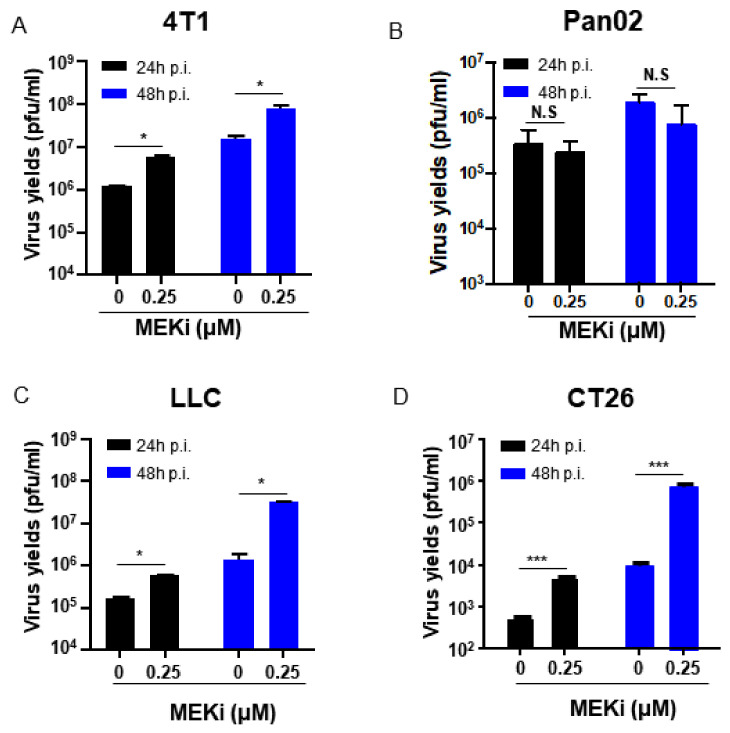
MEKi treatment increased oHSV virus yields in KRAS mutated cancer cells. 4T1 (**A**), Pan02 (**B**), LLC (**C**), and CT26 (**D**) cells were pretreated with 0.25 μM of Trametinib (MEKi) for 2 h and then infected with 0.1 PFU/cell T3855. The virus-containing samples were harvested at 24 and 48 h post-infection, respectively. Additionally, then the titration was measured by conventional plaque assay on Vero cells after three freeze–thaw cycles. N.S. (not significant); *p* > 0.05, * *p* < 0.05, *** *p* < 0.001.

BRAF and KRAS are two key oncogenes in the RAS/RAF/MEK/ERK signaling pathway [18]. Single mutation or concomitant mutations in both KRAS and BRAF genes have been identified in different type of cancers [19,20]. All the three cell lines (Caco-2, Widr, and HT29 cells) with different BRAF mutations mentioned in the above studies, are KRAS wild type cells (Table 1). Then, we sought to determine whether the MEKi treatment can enhance the oHSV replication in the tumor cells with BRAF wt and the upstream KRAS mutations. Thus, the tumor cells with KRAS wt 4T1 cell, Pan02 cell, KRAS G12C-mutated LLC cell, and KRAS G12D-mutated CT26 cells were used (Table 1). The oHSV T3855 containing murine IL-12 and PD-1 antibody, which will be used in the following in vivo study, was used in the combined treatment. As shown in Figure 4C,D, oHSV replication remarkably increased when treated with Trametinib at the concentration of 0.25 μM in KRAS mutated LLC cell (*p* < 0.05) and CT26 cell (*p* < 0.001), while KRAS wt Pan02 cells showed slight decrease of viral replication. In KRAS wt 4T1 cell, the viral replication increased significantly with the same treatment, which may be due to the ERK phosphorylation in 4T1 cells [21] (Figure 4A). It suggested that MEKi Trametinib treatment preferred to enhance oHSV replication in KRAS mutated tumor cells compared with KRAS wt.

**Figure 5:**
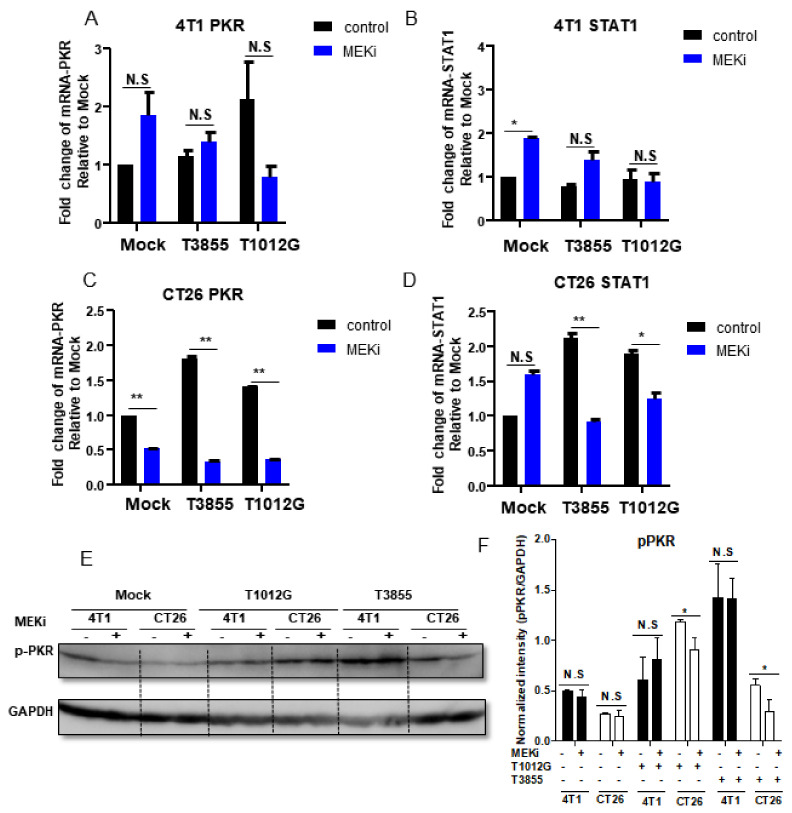
Decreased STAT1 and PKR expression in MEKi treated KRAS mutated cancer cells. 4T1 and CT26 cells were mock treated or pretreated with 0.25 μM of Trametinib (MEKi) for 2 h and then mock infected or infected with 0.1 PFU/cell T1012G or T3855. The cell pellet was harvested at 24 h post-infection, respectively. The total RNAs were extracted and 0.5 μg of RNAs were reverse transcribed to cDNA as described in Materials and Methods. The PKR and STAT1 mRNAs were quantified and normalized with respect to 18S rRNA and shown as fold change compared with mRNA from mock treated and infected cells (Panel (**A**–**D**)). With the same treatment, cell pellets were harvested and the proteins were electrophoretically separated in a 10% denaturing gel and reacted with indicated antibodies (Panel (**E**)). Quantification of the protein level of pERK in 4T1 and CT26 cells (Panel (**F**)). N.S. (not significant); *p* > 0.05, * *p* < 0.05, ** *p* < 0.01.

Next, we sought to investigate whether the augment of oHSV replication was induced by down-regulation of PKR and STAT1 expression when combined treatment with Trametinib. For both oHSVs, T1012G and T3855, we observed similar mRNA expression pattern of PKR and STAT1 during the combined treatment (Figure 5). The result shown in Figure 5A,E,F indicated that the mRNA and phosphorylation level of PKR decreased significantly in MEKi alone and combined treatment in CT26 cells, whereas there was no obvious difference of PKR mRNA and phosphorylation in 4T1 cells with the same treatment (Figure 5C,E,F). Additional studies on the expression of STAT1 showed that combined treatment suppressed STAT1 mRNA expression in CT26 cells but not in 4T1 cells (Figure 5B,D). In addition, there was no STAT1 phosphorylation detected, which may be due to the level of STAT1 phosphorylation in these two murine tumor cell lines. In summary, MEKi Trametinib treatment facilitated oHSV replication, which may be mediated by down-regulation of PKR phosphorylation in KRAS mutant cancer cells.

### 3.5. Combined Treatment with MEKi Trametinib and oHSV T3855 Further Enhanced Anti-Tumor Therapeutic Activity

**Figure 6:**
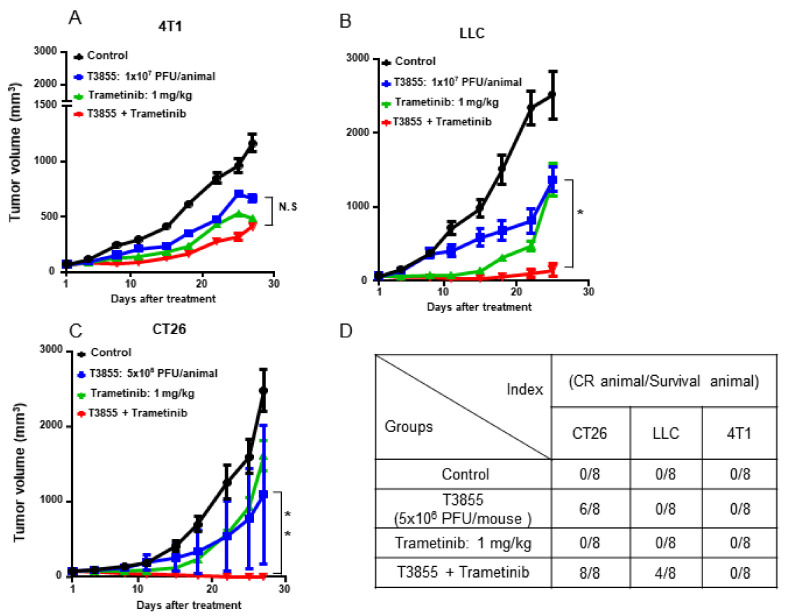
Combined treatment with MEKi Trametinib and oHSV T3855 enhanced antitumor therapeutic activity in LLC and CT26 tumor models. 4T1 (Panel (**A**)) or CT26 (Panel (**C**)) tumor cells were injected s.c. into the right flanks of Balb/c mice, respectively. LLC (Panel (**B**)) tumor cells were injected s.c. into the right flanks of C57BL/6 mice. Different Tumor models averaging 80 mm^3^ (*n* = 8 per group) were treated via intratumoral injection with PBS or T3855 (1 × 10^7^ PFU/animal for 4T1 or LLC; 5 × 10^6^ PFU /animal for CT26) on days 1, 8, 15, and 22 and MEKi (trametinib; 1 mg/kg) or vehicle was given from day 1 to 14 via oral gavage. Tumor volumes are shown as mean ± SEM of 8 animals in each group. The complete tumor eradication (Panel (**D**)) was summarized at the end of the experiment. N.S. (not significant); *p* > 0.05, * *p* < 0.05, ** *p* < 0.01.

Last, whether MEKi Trametinib and oHSV T3855 combination therapy had an enhanced anti-tumor therapeutic efficacy in vivo was investigated. Syngeneic tumor mice were used to implant with murine tumors 4T1, LLC, and CT26, respectively. As shown in Figure 6B,C, delayed tumor growth was observed with Trametinib alone and oHSV T3855 alone, but combined treatment was associated with a significant decrease in tumor growth compared with oHSV alone in LLC model (*p* < 0.05) and CT26 model (*p* < 0.01). Furthermore, 8 of 8 mice were shown complete tumor eradication in the group with combined treatment with CT26 model whereas in LLC model, 4 of 8 mice were shown complete response after 4 weeks treatment (Figure 6D). In addition, we also observed the reductions in tumor volumes in 4T1 model with different treatments compared with control, but there was no significant difference between combined treatment and Trametinib alone (Figure 6A; *p* > 0.05). There were no signs of toxicity or weight loss in any of these animals (data not shown).

## 4. Discussion

Considering the limitation of OVs monotherapy in some tumors, combining OVs with molecular therapeutics targeting kinases associated with cancer development is becoming a promising anti-tumor therapy [4]. In this study, MEK inhibitor Trametinib, was used to combine with oHSV and we provide evidence that pretreatment with Trametinib enhanced oHSV replication mediated by down-regulation of STAT1 and PKR phosphorylation in vitro. Previous studies have described the effects of MAPK pathway inhibition on several oncolytic viruses [22,23]. MEKi CI1040 was found to increase oncolytic adenovirus replication possibly by up-regulation of the expression of coxsackievirus and adenovirus receptor (CAR) in colorectal carcinoma cell [24]. In contrast, MEKi PD98059 increased cell killing, without increasing oncolytic adenovirus replication in glioma cells [25]. For oHSV, combination of T-VEC and MEKi Trametinib increased viral replication in melanoma cell whereas MEK inhibition with PD98059 reduced oHSV-1 R3616 replication by about 15-fold in vitro [26]. It suggested that there is a diverse impact of MEK inhibition on different oncolytic viruses. Furthermore, we have confirmed that the increase of virus replication when pretreated with MEKi Trametinib is correlated with down-regulation of STAT1 and PKR phosphorylation, which likely explains the combination of oHSV-1 and MEKi Trametinib facilitated virus replication.

The first STAT family member, STAT1, primarily mediates cytokine- and growth factor-induced signals that are activated by diverse biological responses including proliferation, differentiation and apoptosis [27]. It has been reported that STAT1 contains highly conserved ERK phosphorylation sites; therefore, STAT protein can be activated by ERK [28,29,30,31]. Here, we further demonstrated that p-STAT1 was significantly down-regulated when decreased levels of p-ERK was induced by MEKi alone and combined. Additionally, the down-regulation of p-PKR was observed with the same treatment. Then, we investigated the possible relevance between STAT1 and PKR when MEKi treated. We found that down-regulation of STAT1 by siRNA can suppress the expression of PKR. Based on these results, we hypothesized that combination of oHSV and MEKi enhanced virus replication possibly mediated by down-regulation of PKR, which is induced by low level of p-STAT1. Interestingly, when mentioned enhancement of oHSV replication with MEKi, it only functions with BRAF V600E-mutated tumor cells, and BRAF wt/RAS-mutated tumor cells, not for BRAF wt/RAS wt tumor cells.

BRAF is a serine-threonine protein kinase involved in the RAS/RAF/MEK/ERK signaling pathway. The most common missense mutation of BRAF (mainly V600E) is known to constitutively activate the MAPK pathway and contributes to the incidence of various cancers [32]. Therapies targeting this pathway, such as those involving a BRAF inhibitor (BRAFi), as well as BRAFi with MEKi have improved antitumor outcomes, although resistance has not been abolished [33]. The combined treatment of oHSV and MEKi performed in this study indicated that combination of MEKi and oHSV increased tumor cell killing by facilitating viral replication, only for BRAF V600E-mutated tumor cells.

In our studies, therapeutic effectiveness was seen in syngeneic tumor mice with BRAF wt/KRAS-mutations. We found that in CT26 (KRAS-G12D) mouse model, the combined treatment of oHSV T3855 with MEKi showed 100% complete tumor eradication while LLC (KRAS-G12C) showed 50% complete response at 4 weeks post treatment. The remarkably therapeutic efficacy may be induced by increased virus replication as we have determined that MEKi treatment enhanced oHSV replication in BRAF wt/KRAS mutated tumor cells in vitro. On the other hand, the oHSV T3855 used in vivo contains murine IL-12 and murine scFV (single-chain variable fragment) antibody against PD-1. The combination of oHSV T3855 with MEKi is equal to a triple-drug regimen, including oncolytic effect of oHSV, PD-1 blockade, and MEK inhibition. It is consistent with the augmented antitumor responses with the combination of T-VEC, MEK inhibition, and anti-PD-1 antibody [9]. In addition, down-regulation of PKR and STAT1 mRNA levels was also observed in KRAS mutant cancer cells when combined treatment performed. It has been reported that in in human KRAS-G12D colon tumor cells, STAT1 can act in a cell-autonomous manner to promote tumor growth [34], which indicated that inhibition of STAT1 will suppress the KRAS-G12D colon tumor growth.

In summary, combination treatment with oHSV and MEKi Trametinib enhanced virus replication mediated by down-regulation of STAT1 and PKR phosphorylation in BRAF V600E-mutated tumor cells as well as BRAF wt/KRAS-mutated tumor cells. In vivo, there is a synergistic therapeutic efficacy shown in the combination of oHSV including PD-1 blockade and MEK inhibition, only for BRAF wt/KRAS-mutated tumors. Collectively, these data provide some new insights for clinical development of combination therapy with oncolytic virus, MEK inhibition, and checkpoint blockade for BRAF V600E-mutated or KRAS-mutated tumors.

## 5. Conclusions

Treatment with MEKi Trametinib augmented oHSV oncolytic activity in BRAF V600E-mutated tumor cells. Combination treatment with oHSV and MEKi Trametinib enhanced virus replication mediated by down-regulation of STAT1 and PKR expression or phosphorylation in BRAF V600E-mutated tumor cells as well as BRAF wt/KRAS-mutated tumor cells. A remarkably synergistic therapeutic efficacy was shown in vivo for BRAF wt/KRAS-mutated tumor models, when a combination of oHSV including PD-1 blockade and MEK inhibition.

## Figures and Tables

**Table 1:** Tumor cells with different BRAF and KRAS mutations.

Cell Line		BRAF	KRAS
Caco-2	Human Colon Carcinoma	wt	wt
Widr	Human Colon Carcinoma	V600E	wt
HT29	Human Colon Carcinoma	V600E	wt
4T1	Mouse Breast Carcinoma	wt	wt
Pan02	Mouse Pancreatic Carcinoma	wt	wt
LLC	Mouse Lung Carcinoma	wt	G12C
CT26	Mouse Colon Carcinoma	wt	G12D

## Data Availability

All data related to this study will be made available upon request.
